# Effects of pre-exercise high and low glycaemic index meals on substrate metabolism and appetite in middle-aged women

**DOI:** 10.1017/jns.2023.96

**Published:** 2023-11-20

**Authors:** Miki Sakazaki, Yoshie Yoshikawa, Kayoko Kamemoto, Yusei Tataka, Yoshiki Yamada, Ching-Lin Wu, Masashi Miyashita

**Affiliations:** 1 Graduate School of Sport Sciences, Waseda University, 2-579-15 Mikajima, Tokorozawa, Saitama 359-1192, Japan; 2 Waseda Institute for Sport Science, Waseda University, 2-579-15 Mikajima, Tokorozawa, Saitama 359-1192, Japan; 3 Graduate Institute of Sports and Health Management, National Chung Hsing University, Taichung 402202, Taiwan; 4 Faculty of Sport Sciences, Waseda University, 2-579-15 Mikajima, Tokorozawa, Saitama 359-1192, Japan; 5 School of Sport, Exercise and Health Sciences, Loughborough University, Loughborough, Leicestershire LE11 3TU, UK; 6 Department of Sports Science and Physical Education, The Chinese University of Hong Kong, Shatin, Hong Kong

**Keywords:** Appetite-related hormones, Fat oxidation, Females, Food reward, Low glycaemic index, Walking

## Abstract

Few studies have examined the influence of pre-exercise meals with different glycaemic indices (GIs) on substrate oxidation and non-homeostatic appetite (i.e. food reward) in adults of various ages and ethnicities. We aimed to examine the effects of pre-exercise high and low GI meals on substrate oxidation and food reward in middle-aged Japanese women. This randomised crossover trial included fifteen middle-aged women (aged 40⋅9 ± 6⋅5 years, mean ± sd). The participants consumed a high or low GI breakfast at 09.00 and rested until 11.00. Thereafter, participants performed a 60-min walk at 50 % of their estimated maximum oxygen uptake (11.00–12.00) and rested until 13.00. Expired gas samples were collected every 30 min prior to walking, and samples were collected continuously throughout the walking and post-walking periods. Blood samples and subjective appetite ratings were collected every 30 min, except during walking. The Leeds Food Preference Questionnaire in Japanese (LFPQ-J) was used to assess food reward at 09.00, 10.00, and 13.00 h. The cumulative fat oxidation during exercise was higher in the low GI trial than in the high GI trial (*P* = 0⋅03). The cumulative carbohydrate oxidation during walking was lower in the low GI trial than in the high GI trial (*P* = 0⋅01). Trial-by-time interactions were not found for any food-reward parameters between trials. Low GI meals elicited enhanced fat oxidation during a subsequent 60-min walk in middle-aged women. However, meals with different GIs did not affect food reward evaluated over time in the present study.

## Introduction

The glycaemic index (GI) is an indicator of the glycaemic response after the ingestion of carbohydrate-containing foods.^(1)^ Glucose is the predominant elevated substrate in the circulation during the postprandial period and hyperglycaemia is a risk factor for cardiovascular disease and all-cause mortality in humans.^(2,3)^ A low GI food has been shown to be effective in controlling glycaemia and cardiometabolic risk factors as one of the dietary approaches for individuals with diabetes.^(4)^ In addition to the use of low GI diets for glycaemic control in clinical populations, low GI foods have also been used to evaluate other health-related outcomes in non-clinical settings. Although the GI of foods does not quantitatively evaluate the rate of glucose disappearance from the circulation, a slow and steady release of glucose into the circulation appears beneficial for substrate oxidation during subsequent exercise^(5)^ or enhanced satiety during subsequent rest^(6)^ in healthy adults, partly because of the lowered postprandial insulin concentration.^(7,8)^ Therefore, an increase in fat oxidation and an enhancement of satiety through dietary manipulation of low GI foods may be of benefit for a better long-term weight management.

Regarding exercise manipulation to maximise the benefits of low GI diets on substrate oxidation during subsequent exercise, a large and consistent body of evidence, but not all,^(9–12)^ shows that a low GI meal consumed prior to an acute bout of exercise reduces glycaemic responses, leading to an increase in fat oxidation and a reduction in carbohydrate oxidation during exercise compared with consumption of a high GI meal.^(13–25)^ Most of the participants in these previous studies,^(10–17,19,20,22–24)^ except for three studies that included young women,^(9,18,21)^ were males ranging in age from young to middle-aged. Given that an impairment of glucose tolerance and insulin clearance, and a decline in basal fat oxidation are associated with aging,^(26–29)^ especially in women, possibly mediated through enhanced postprandial fat storage in adipose tissue despite having a higher lipolytic activity during exercise than in men,^(30,31)^ investigations in middle-aged or older women may be of importance. Furthermore, limited information is available on the effects of pre-exercise with different GI meals on substrate metabolism in Asians^(24)^ as most previous studies have been conducted on Caucasians. Investigations in other ethnicities represent an important gap in the current understanding, considering that excursions in glucose and insulin concentrations after meals differ among ethnic groups, even among Asian populations.^(25)^

The acute effects of pre-exercise GI meal(s) on subjective appetite^(16–19,21,32)^ and appetite-related hormones^(21)^ have been evaluated in previous laboratory-based studies, and the findings were inconsistent. Some studies have shown that the consumption of a low GI meal results in enhanced fullness only during the postprandial period compared with a high GI meal,^(17,18)^ whereas other studies reported no differences in the feeling of fullness^(16,19,21,32)^ or appetite-related hormones.^(21)^ Previous studies have only examined homeostatic appetite and none have addressed non-homeostatic appetite after the consumption of different GI meals. The latter appetite is modulated by the food-reward system, which is manifested by ‘liking’ and ‘wanting’ for specific foods, and provides direction and intensity to the motivation to eat.^(33)^ It has been reported that the magnitude of change in insulin concentration may attribute to these reward behaviours.^(34)^ This issue is important to address because such an evaluation enables us to understand the qualitative aspects of appetite in the context of food preference and rewards through dietary and exercise manipulations.

Therefore, the present study aimed to investigate the effects of pre-exercise meals with high and low GI values on substrate oxidation and non-homeostatic appetite in middle-aged Japanese women. We hypothesised that fat oxidation during exercise would increase and the preference for high fat foods would be suppressed following the consumption of a low GI meal compared to a high GI meal (i.e. before exercise).

## Methods

### Participants

This study was registered in advance with the University Hospital Medical Information Network Center (UMIN), a system for registering clinical trials (ID: UMIN000046210; URL: https://center6.umin.ac.jp/cgi-open-bin/ctr_e/ctr_view.cgi?recptno = R000052489). This study was conducted according to the guidelines laid down in the Declaration of Helsinki^(35)^ and all procedures involving human participants were approved by the Ethics Committee on Human Research of Waseda University (approval number: 2021-306). Written informed consent was obtained from all nineteen participants. They were Japanese (i.e. self-reported ethnicity) healthy, middle-aged women. None of the participants were having habits of continued ‘exercise’ for at least 30 min/d and at least 2 d/week for at least a year.^(36)^ However, their activities of daily living evaluated by a questionnaire were relatively active through their occupational and household works. The exclusion criteria of the present study were as follows: age < 30 or >59 years, taking any medication or supplement known to affect substrate metabolism, major illness, current smoker, and body mass that had not been stable for at least 3 months before the study or intention to lose weight during the study, participation in other studies while participating in the present study, history of an immediate allergic reaction to meals (i.e. each food item provided as a test meal in the present study), pregnancy and suspected pregnancy or within a year of parturition.

### Study design and experimental protocol

**Fig. 1.**
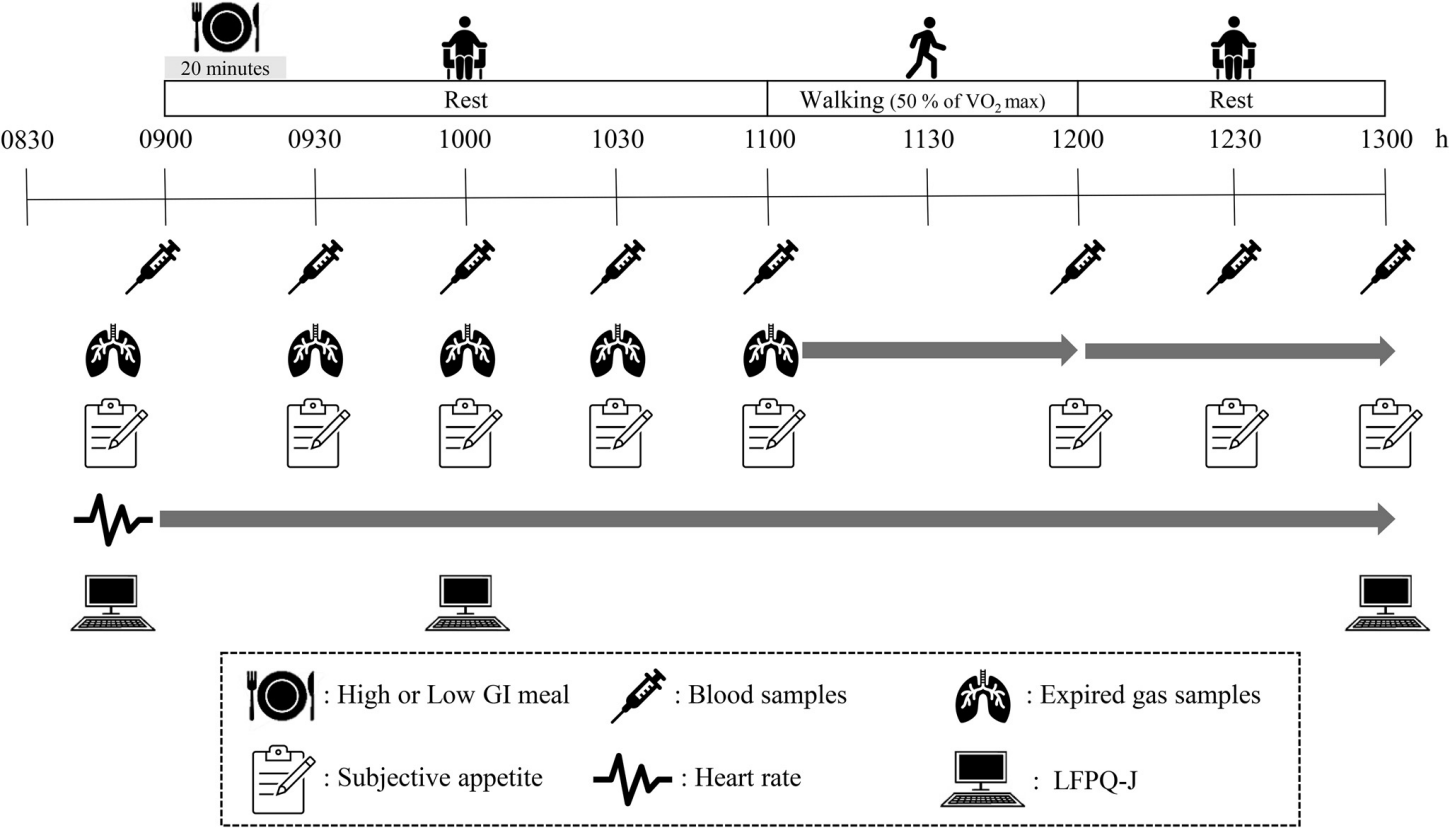
Schematic illustration of the study protocol. GI, glycaemic index; VO_2_max, maximum oxygen uptake; LFPQ-J, Leeds Food Preference Questionnaire in Japanese.

Participants completed two, 1-day laboratory-based trials in random order: a high GI trial and a low GI trial. The lead investigator enrolled the participants in the research and randomly assigned the participants to each experiment in a counterbalanced manner using computer-generated random numbers. The interval between trials was at least 7 d. All trials were conducted during the follicular phase of the menstrual cycle (days 1–10), based on the self-reported onset of menstruation. A schematic illustration of the study protocol is shown in Fig. 1.

In both trials, all participants reported to the laboratory at 08.30 after a 10-h overnight fast (no food or drink, except water). Body mass was measured to the nearest 0⋅1 kg using a digital scale (MC-780A-N, Tanita Corporation, Tokyo, Japan). A heart rate monitor (Polar RCX3, Polar Electro, Kempele, Finland) was then fitted to measure heart rate continuously throughout the trial. After a 10-min seated rest, subjective appetite using a paper-based questionnaire (details in ‘Subjective appetite’), food reward using the LFPQ-J (details in ‘Food reward’), and baseline measurements of a 15-min resting expired gas sample were obtained sequentially. Thereafter, a cannula was inserted into the arm vein, and a fasting venous blood sample was collected in a seated position. The participants then consumed a high or low GI meal at 09.00. Participants were asked to consume the test meal over 20 min (i.e. the last bite was set at 09.20), and the order of food intake in each meal was standardised. Subsequently, they were required to rest for 100 min before performing the exercise. During the 60-min walking exercise on the treadmill at a speed eliciting 50 % of the estimated maximum oxygen uptake (determined from the preliminary test) (11.00–12.00), oxygen uptake, respiratory exchange ratio (RER), fat oxidation rate, and carbohydrate oxidation rate were measured using a stationary gas analyser (Quark RMR, COSMED Co. Ltd., Roma, Italy), and RPEs were assessed periodically.^(37)^ The participants were then asked to sit on a chair in a comfortable position for 60 min from 12.00 to 13.00. Further expired gas samples were collected every 30 min for 15 min prior to walking and were collected continuously throughout the walking and post-walking periods in both trials. Venous blood samples were collected using a cannula to measure the circulating concentrations of glucose, insulin, triglyceride (TG), non-esterified fatty acids (NEFA), glycerol, 3-hydroxybutyrate, total peptide tyrosine tyrosine (PYY), acylated ghrelin, and subjective appetite every 30 min, except during walking, throughout the trial. Furthermore, food rewards using the LFPQ-J were evaluated at 10.00 and 13.00 in both trials. The participants consumed water *ad libitum* during the first trial, and the volume ingested was replicated in subsequent trials. Average water intake was 366⋅3 ± 143⋅0 ml over 4 h.

### Anthropometry

Body mass and body fat percentage were measured to the nearest 0⋅1 kg and 0⋅1 %, respectively, using a digital scale (TANITA MC780, Tanita Corporation, Tokyo, Japan). Height was measured using a stadiometre (YS-OA, Yoshida Seisakusho Ltd., Gifu, Japan) to the nearest 0⋅1 cm. Body mass index was calculated by dividing body mass in kilogrammes by the square of height in metres.

### Screening and preliminary tests

Following the anthropometric assessments, the Japanese version of the Three Factor Eating Questionnaire (TFEQ)^(38)^ was conducted (details in ‘Three Factor Eating Questionnaire’). Thereafter, a screening test of the Leeds Food Preference Questionnaire in Japanese (LFPQ-J), which was developed specifically for the Japanese population, was conducted^(39)^ (details in ‘Food reward’). The purpose of the screening test was to ask participants about the names of the sixteen foods used in the LFPQ-J, their allergies, whether they had ever eaten them, and whether they could eat them. After the screening test, participants practiced the tasks performed on the LFPQ-J. Then, resting heart rate was recorded for 1 min after 5 min of seated resting using a short-range telemetry (Polar RCX3, Polar Electro, Kempele, Finland). After familiarisation with the treadmill (JOG NOW 700, Technogym, Cesena, Italy), each participant was asked to perform a submaximal preliminary exercise test. This test consisted of a 16-min, four-stage incremental walk test to establish the relationship between treadmill speed and oxygen uptake, or heart rate. The initial walking speed was set to 4⋅0 km/h and was increased by 1⋅0 km/h every 4 min. The treadmill inclination was set to 0 % throughout the test. Oxygen uptake, carbon dioxide production, and RER were measured breath-by-breath using a stationary gas analyser (Quark RMR, COSMED, Rome, Italy). Heart rate was measured continuously using a short-range telemetry (Polar RCX3, Polar Electro, Kempele, Finland). Ratings of perceived exertion (RPEs) were recorded during the final 15 s of each stage using the Borg scale.^(37)^ Data from the preliminary exercise test were used to estimate the walking workload (i.e. walking speed) corresponding to 50 % of each participant's estimated maximum oxygen uptake, and this workload was used for the main trials.

### Three Factor Eating Questionnaire

The 51-item TFEQ is a self-assessment tool that measures eating behaviour and consists of three factors: cognitive restraint, disinhibition, and hunger.^(40)^ The Japanese version of the TFEQ^(38)^ was used to assess participants’ eating behaviour traits in the present study. Although no clear criteria were provided, a score of 14 for cognitive restraint, 14 for disinhibition, and 7 for hunger were considered elevated.^(40)^

### Standardisation of dietary intake and physical activity

The participants weighed and recorded all the food and drinks consumed the day before each main trial and refrained from drinking alcohol during this period. They replicated their energy intake from the first to the second trial to ensure that it was standardised across trials. Food diaries were analysed using a nutrition analysis software (Excel Eiyoukun Ver 9.0, Kenpakusha, Tokyo, Japan) by a registered dietician to determine the energy intake and macronutrient content of the foods. Participants were asked to avoid strenuous exercise for 1 d before each main trial. They wore a uniaxial accelerometer (Lifecoder-EX, Suzuken Co. Ltd., Nagoya, Japan) on their hips to objectively monitor their daily activities during this period. The reliability of this accelerometer was validated against whole body indirect calorimetry.^(41)^ The accelerometer defined eleven levels of activity intensity (0, 0⋅5, and 1–9), with 0 indicating the lowest intensity and 9 indicating the highest intensity. A level of 4 corresponds to an intensity of approximately 3 metabolic equivalents.^(41)^ Levels 1–3 correspond to light physical activity, levels 4–6 correspond to moderate physical activity, and levels 7–9 correspond to vigorous physical activity. On the day before each main trial, the participants received text messages from a researcher asking them to replicate their energy intake and physical activity patterns. Their compliance with replicating each main test condition was verified upon arrival at the laboratory during the main trials.

### Test meals

The contents of the high GI and low GI meals are presented in Table 1. Briefly, the high GI meal consisted of corn flakes containing granulated sugar, skim milk, white bread, margarine, instant mashed potatoes, and carbonated drinks (GI = 73). The low GI meal comprised brown rice with seasoning, skim milk, yogurt, canned peaches, cherry tomatoes, and apple juice (GI = 41). The energy density of the test meal was 5⋅4 and 2⋅9 kJ/g for the high and low GI meals, respectively. The high and low GI meals were adjusted to contain the same energy (33 kJ/kg body mass) and macronutrient content (1⋅5 g/kg body mass for carbohydrate, 0⋅1 g/kg body mass for fat, and 0⋅3 g/kg body mass for protein). Individual GI values for the foods in the meals were adapted from the International Table of Glycaemic Index and Glycaemic Load Values 2002^(42)^ and 2008.^(43)^ The GI of all meals was calculated from the weighted mean of the GI values for the food components.^(44)^

**Table 1.** The nutritional contents of the high and low glycaemic index (GI) meals (for a 50 kg participant)

	Quantity	Energy	CHO	Fat	Protein	Fibre
	(g)	(kJ)	(g)	(g)	(g)	(g)
Food item						
High GI meal						
Corn flakes	26⋅0	410	22⋅6	0⋅3	2⋅0	0⋅7
Granulated sugar	5⋅0	79	5⋅0	0⋅0	0⋅0	0⋅0
Skim milk	160⋅0	251	8⋅5	0⋅2	6⋅2	0⋅0
White bread	30⋅0	351	16⋅0	1⋅0	2⋅9	0⋅6
Instant mashed potato	20⋅0	310	16⋅6	0⋅2	1⋅5	1⋅3
Carbonated drink	67⋅0	117	6⋅7	0⋅0	0⋅0	0⋅0
Margarine	5⋅0	126	0⋅0	3⋅3	0⋅0	0⋅0
Total	313⋅0	1644	75⋅3	4⋅9	12⋅4	2⋅5
GI	73^a^					
Low GI meal						
Brown rice	75⋅0	456	24⋅0	1⋅0	1⋅7	1⋅2
Seasoning	1⋅8	29	0⋅3	0⋅3	0⋅8	0⋅0
Skim milk	130⋅0	247	6⋅9	0⋅1	5⋅0	0⋅0
Yogurt	120⋅0	310	6⋅4	3⋅6	4⋅1	0⋅0
Canned peaches	120⋅0	360	21⋅0	0⋅4	0⋅5	1⋅7
Apple juice	120⋅0	226	13⋅2	0⋅0	0⋅1	0⋅0
Cherry tomato	45⋅0	59	3⋅2	0⋅0	0⋅5	0⋅5
Total	611⋅8	1644	74⋅9	5⋅3	12⋅7	3⋅3
GI	41^a^					

CHO, carbohydrate.

a Calculated by the method previously described^(44)^ with GI values from the International Table of Glycaemic Index and Glycaemic Load Values: 2002^(42)^ and 2008.^(43)^

### Blood collection and analysis

For plasma glucose measurements, blood samples were collected in tubes containing sodium fluoride-EDTA (Venoject 2, Terumo Corporation, Tokyo, Japan) and stored at 4 °C for later analysis. For plasma insulin, total PYY, and glycerol measurements, venous blood samples were collected in dipotassium salt-EDTA tubes (Venoject 2, Terumo Corporation, Tokyo, Japan) and were centrifuged immediately at 1861 *g* for 10 min at 4 °C. Plasma samples were then removed, aliquoted and stored at −80 °C for later analysis. For plasma-acylated ghrelin measurements, blood samples were immediately transferred to EDTA tubes containing aprotinin (Neo tube, Nipro Corporation, Osaka, Japan) to prevent degradation of ghrelin by proteases and were immediately centrifuged as described above. For serum TG, NEFA, 3-hydroxybutyrate, total cholesterol (Total-C), low-density lipoprotein cholesterol (LDL-C), and high-density lipoprotein cholesterol (HDL-C) measurements, venous blood samples were collected in tubes containing clotting activators for serum isolation (Venoject 2, Terumo Corporation, Tokyo, Japan). The samples were allowed to clot for 30 min at approximately 22–23 °C and then centrifuged at 1861 *g* for 10 min at 22 °C. Thereafter, the samples were stored at 4 °C for later analysis. Enzymatic colourimetric assays were used to measure plasma glucose (GLU-HK(M), Shino-Test Corporation, Kanagawa, Japan), serum TG (Pure Auto S TG-N, Sekisui Medical Co. Ltd., Tokyo, Japan), serum NEFA (NEFA-HR, Wako Pure Chemical Industries Ltd., Osaka, Japan), serum 3-hydroxybutyrate (KAINOS 3-HB, Kainos Laboratories, Inc., Tokyo, Japan), serum Total-C (Total-C Determiner L TC II, Hitachi Chemical Diagnostics Systems Co Ltd., Tokyo, Japan), serum LDL-C (Matabo Lead LDL-C, Hitachi Chemical Diagnostics Systems Co Ltd., Tokyo, Japan), and serum HDL-C (Matabo Lead HDL-C, Hitachi Chemical Diagnostics Systems Co Ltd., Tokyo, Japan). Enzyme-linked immunosorbent assays (ELISA) were used to measure plasma insulin (Mercodia Insulin ELISA, Mercodia AB, Uppsala, Sweden), total PYY (YK080, Yanaihara Institute Inc., Shizuoka, Japan), plasma glycerol (Glycerol Colorimetric Assay Kit, Cayman Chemical Company, MI, USA), and acylated ghrelin (A05306, Bertin Pharma, Montigny-le-Bretonneux, France). The intra-assay coefficients of variation were 0⋅7 % for glucose, 2⋅2 % for insulin, 1⋅2 % for TG, 0⋅7 % for NEFA, 1⋅3 % for 3-hydroxybutyrate, 0⋅7 % for Total-C, 1⋅0 % for LDL-C, 1⋅2 % for HDL-C, 4⋅2 % for glycerol, 3⋅9 % for total PYY, and 7⋅5 % for acylated ghrelin.

### Food reward

Food reward was measured using the LFPQ-J, a computer-based task that assesses the different components of food preference and reward.^(39)^ The LFPQ-J measures explicit liking and wanting directly and implicit wanting indirectly, using sixteen images of foods that are either high fat savoury, low fat savoury, high fat sweet, or low fat sweet. The sixteen food images used in the present study are presented in Supplementary Table S1.

The LFPQ-J consists of two tasks: single- and paired-food tasks. Food reward was evaluated using the following four parameters: explicit liking, explicit wanting, implicit wanting, and relative preference. In the single-food task, participants were asked to rate the extent to which they thought they were liking or wanting each randomly presented food item on a 100-mm visual analogue scale to measure explicit liking and wanting. Briefly, single-food images were randomly shown to the participant, and participants responded according to the following two questions: ‘How pleasant would it be to taste some of this food now?’ (explicit liking) and ‘How much do you want some of this food now?’ (explicit wanting), anchored at each end with ‘not at all’ and ‘extremely’. In the paired-food trials, each food image was presented in turn with a food image from the other category, and participants were instructed to select the food they ‘most want to eat now’ as quickly as possible. Implicit wanting was measured using the reaction time of the test and selected categories, and relative preference was measured using the number of selections per category.^(45)^ Briefly, a series of food image pairs were presented to participants, who were asked, ‘Which food do you most want to eat now?’. These food pairs were presented in ninety-six pairs, such that all food images from one category were presented with each food from the other categories. The participants were asked to select the food they wanted to eat the most at that moment as quickly and accurately as possible using a keyboard press. The exclusion criteria for response time were shorter than 100 ms or longer than 8000 ms. The frequency of choice and non-choice and the reaction time of each task for each food category were recorded, and the implicit wanting score was calculated using the following formula.^(45)^ A positive score indicates a greater preference for a given food category relative to the alternatives in the task, whereas a negative score indicates the opposite. A score of zero indicates that the category is equally preferred.
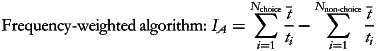
where *I_A_* denotes implicit wanting for category *A*; *N*_choice_ denotes the number of times category *A* was selected; *N*_non-choice_ denotes the number of times category *A* was not selected; and 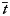 denotes the mean of all reaction times.^(45)^

For the parameters measured in all food-reward measurements, the fat appeal bias for foods with different fat contents and the taste appeal bias for foods with different tastes were evaluated. Bias scores for fat content and taste were computed by subtracting the mean low fat scores from the mean high fat scores and the mean savoury scores from the mean sweet scores. Positive values indicate a preference for high fat and/or sweet foods; negative values indicate a preference for low fat and/or savoury foods; and a score of 0 indicates an equal preference between fat content and taste categories.^(45)^

### Subjective appetite

Subjective appetite (satiety, fullness, hunger, and prospective food intake) was assessed on a 100-mm visual analogue scale using a paper-based questionnaire (each end of the line represents the most extreme sensation experienced by the participant).^(46)^ The overall subjective appetite score was calculated from the results of the four appetite ratings using the following equation: Satiety + fullness + (100 – hunger) + (100 – prospective food intake)/4,^(47)^ where 100 indicates low appetite and 0 indicates high appetite.

### Calculations and statistical analysis

We calculated the required sample size based on data from a previous study^(18)^ using G*Power 3.1.9.6.^(48)^ The previous study reported a between-trial difference in the total amount of fat oxidation throughout the exercise (10⋅4 ± 6⋅5 g/h, mean ± sd) between the high GI trial and the low GI trial.^(18)^ Based on this study with eight participants, we calculated that an estimated total sample size of 11 was needed to provide 80 % power to detect between-trial differences, with an alpha level set at 0⋅05 and a correlation of 0⋅5. This sample size estimation was powered to detect an effect size (ES) of 0⋅94 (Cohen's *d*), using a paired *t*-test for comparisons between the two trials. Based on this calculation, nineteen participants were recruited to allow for potential withdrawals. Data were analysed using IBM SPSS Statistics for Windows version 28.0 (IBM Corp., NY, USA). The rates of fat and carbohydrate oxidation and gross energy expenditure were estimated from oxygen uptake and carbon dioxide production using stoichiometric equations.^(49)^ Total fat and carbohydrate oxidation during the 60-min walking exercise was estimated from the cumulative rate of oxidation for each participant. The total and incremental area under the curve (AUC) were calculated using GraphPad Prism version 9.2.0 for Windows (GraphPad Software Inc., CA, USA). Generalised estimating equations were used to examine the between-trial differences for all parameters. Where values were different at baseline, generalised estimating equations were used to adjust for baseline values when examining differences over time between trials. Where a significant trial-by-time interaction was found, *post-hoc* pairwise comparisons were performed using the Bonferroni method. Where statistically significant differences in baseline values were found, statistical analysis was performed by adjusting for baseline covariates. The 95 % confidence intervals (95 % CI) for the mean absolute pairwise differences between trials were calculated using the *t*-distribution and degrees of freedom (*n*–1). Effect sizes (Cohen's *d*) were calculated to describe the magnitude of differences between trials. Effect sizes of 0⋅2 are considered the minimum important differences in all outcome measures, 0⋅5 moderate, and 0⋅8 large.^(50)^ Statistical significance was accepted at the <5 % level. Results are reported as mean ± sd.

## Results

### Participants

**Fig. 2.**
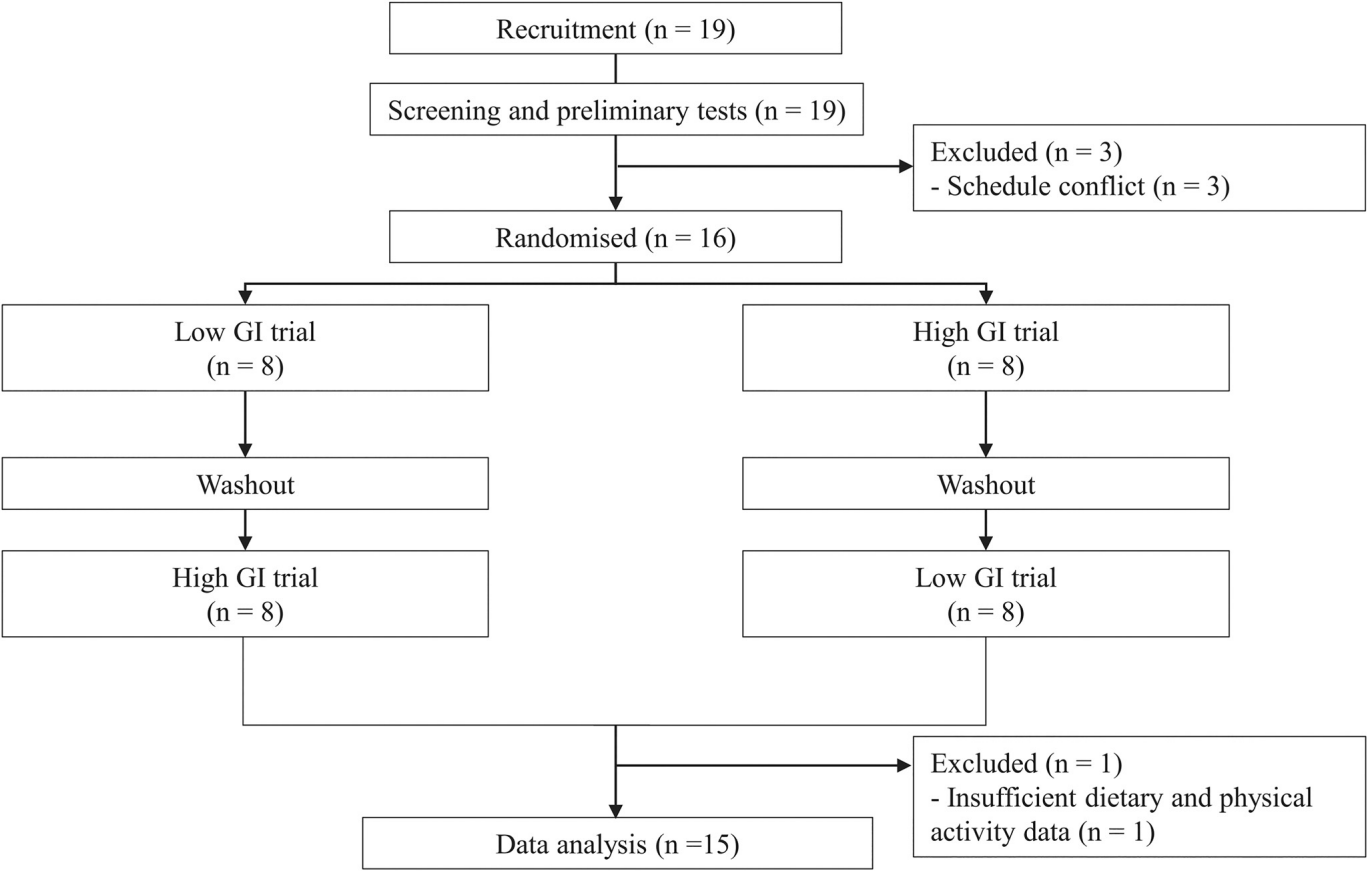
A participant flow diagramme. GI, glycaemic index.

The participant flow diagramme is shown in Fig. 2. After nineteen participants signed the informed consent, three dropped out of the study before starting the first main trial because of a schedule conflict. Sixteen participants were randomly assigned to the intervention and completed two trials as described in the methods section. However, one participant did not follow the prescribed instructions for standardisation of dietary intake and physical activity. Therefore, fifteen participants were included in the data analysis. The descriptive characteristics of the participants included in the data analysis were as follows: age 40⋅9 ± 6⋅5 years, height 1⋅59 ± 0⋅04 m, body mass 52⋅1 ± 4⋅3 kg, body mass index 21 ± 1 kg/m^2^, body fat 15⋅0 ± 4⋅4 %, and estimated maximum oxygen uptake of 34⋅4 ± 6⋅8 ml/kg/min.

### Physical activity, dietary record data, and eating behaviour

The accelerometer-recorded physical activity level of the participants for the day prior to the two trials did not differ (total step counts, high GI: 9783 ± 4264 *v.* low GI: 9899 ± 4539 steps/d, 95 % CI −814⋅3 to 582⋅1 steps/d, *P* = 0⋅74; light physical activity, high GI: 62⋅3 ± 30⋅2 *v.* low GI: 56⋅6 ± 31⋅4 min/d, 95 % CI −5⋅7 to 17⋅0 min/d, *P* = 0⋅33; moderate physical activity, high GI: 33⋅1 ± 18⋅8 *v.* low GI: 33⋅8 ± 25⋅5 min/d, 95 % CI −10⋅7 to 9⋅8 min/d, *P* = 0⋅93; vigorous physical activity, high GI: 5⋅0 ± 6⋅0 *v.* low GI: 3⋅1 ± 3⋅0 min/d, 95 % CI −1⋅0 to 4⋅8 min/d, *P* = 0⋅14). All the participants reported that they had consumed identical foods and drinks on the day before the two trials. The mean self-reported energy intake for the day prior to each trial was 7⋅1 ± 1⋅3 MJ/d (57⋅5 ± 7⋅9 % from carbohydrate, 13⋅8 ± 3⋅9 % from protein, and 28⋅7 ± 6⋅9 % from fat). Results from the Japanese version of the TFEQ were as follows: cognitive restraint, 9⋅1 ± 3⋅6; disinhibition, 5⋅7 ± 3⋅5; and hunger, 3⋅7 ± 2⋅8.

### Oxygen uptake, energy expenditure, heart rate, and RPE during walking

There were no significant differences in oxygen uptake (high GI: 0⋅88 ± 0⋅18 (corresponding to 49⋅4 ± 5⋅6 % of estimated maximum oxygen uptake) *v.* low GI: 0⋅86 ± 0⋅13 (corresponding to 48⋅8 ± 4⋅8 % of estimated maximum oxygen uptake) l/min, 95 % CI −0⋅01 to 0⋅04 l/min, *P* = 0⋅28), gross energy expenditure (high GI: 1⋅0 ± 0⋅2 *v.* low GI: 1⋅0 ± 0⋅2 MJ, 95 % CI −0⋅02 to 0⋅07 MJ, *P* = 0⋅26), or RPEs (high GI: 11 ± 2 *v.* low GI: 11 ± 2, 95 % CI −0⋅3 to 0⋅2, *P* = 0⋅86) during the 60-min walk between trials. The mean heart rate was higher in the high GI trial than the low GI trial (high GI: 112 ± 13 *v.* low GI: 110 ± 11 beats/min, 95 % CI 0⋅8 to 3⋅5 beats/min, *P* = 0⋅002, ES = 0⋅32).

### Substrate oxidation

**Fig. 3.**
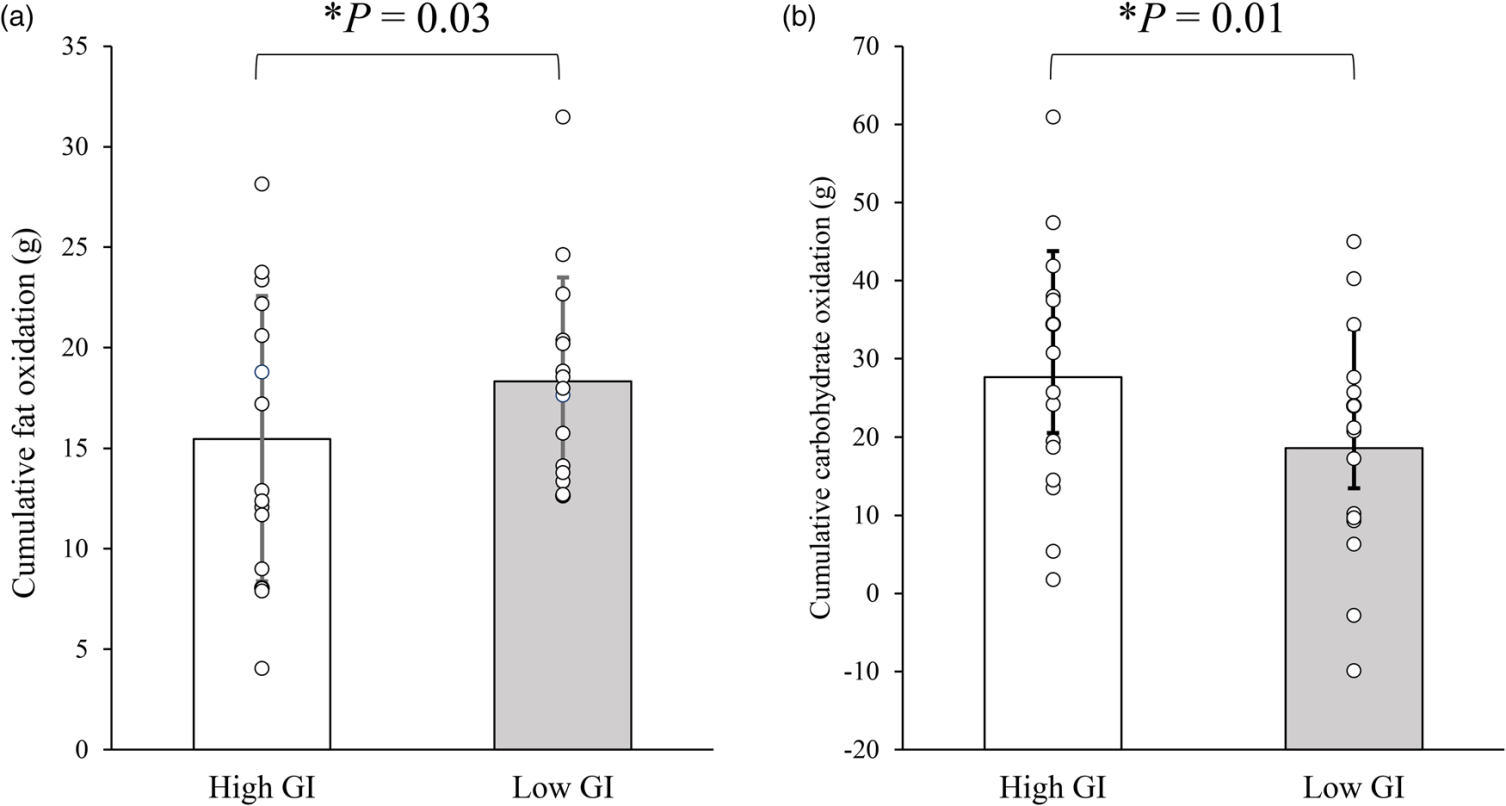
Cumulative fat (a) and carbohydrate oxidation (b) during the 60-min walking exercise in the high and low glycaemic index (GI) trials. Values are means ± sd. Values were compared using generalised estimating equations. *Post-hoc* analysis was adjusted for multiple comparisons using the Bonferroni method. *Significantly different between trials.

The cumulative fat and carbohydrate oxidation values during the 60-min walk (11.00–12.00) are shown in Fig. 3. The cumulative fat oxidation was higher in the low GI trial than in the high GI trial (high GI: 15⋅5 ± 7⋅1 *v.* low GI: 18⋅3 ± 5⋅2 g, 95 % CI 0⋅3 to 5⋅4 g, *P* = 0⋅026, ES = 0⋅56; Fig. 3(a)). The cumulative carbohydrate oxidation was lower in the low GI trial than in the high GI trial (high GI: 27⋅6 ± 16⋅2 *v.* low GI: 18⋅6 ± 15⋅2 g, 95 % CI −16⋅0 to −1⋅0 g, *P* = 0⋅01, ES = 0⋅63; Fig. 3(b)).

**Fig. 4.**
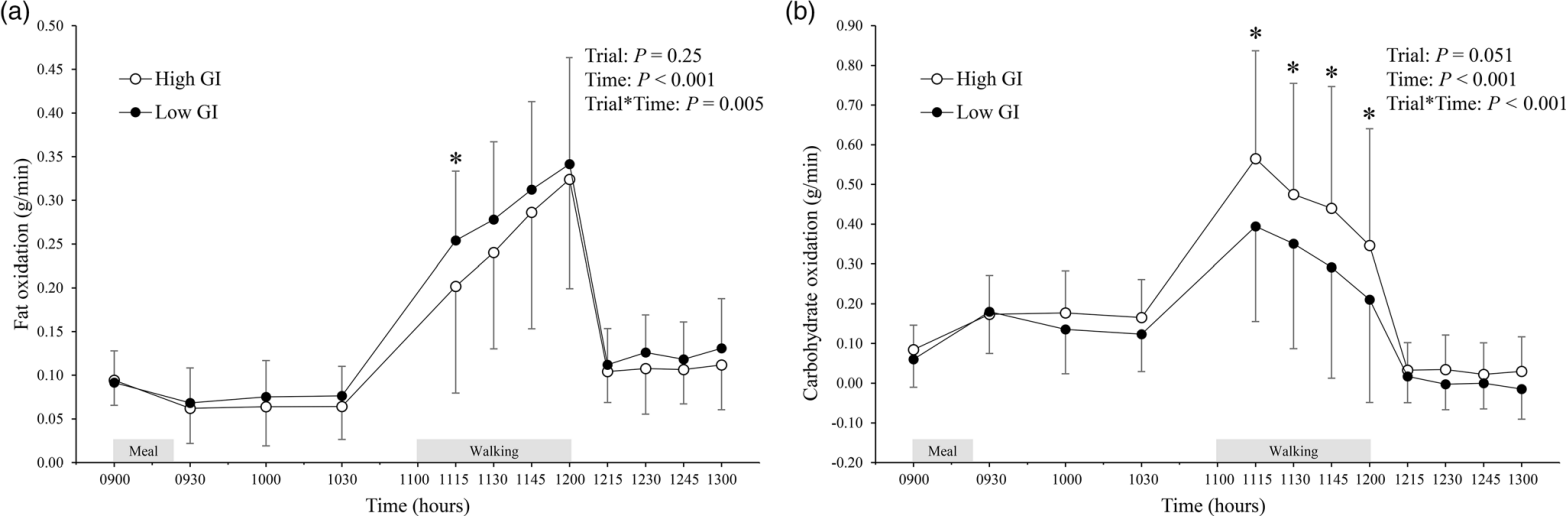
Fat (a) and carbohydrate oxidation rate (b) in the high and low glycaemic index (GI) trials. Values are means ± sd represented by unidirectional bars. Values were compared using generalised estimating equations. *Post-hoc* analysis was adjusted for multiple comparisons using the Bonferroni method. *Significantly different between trials at the same time point, *P* = 0⋅03 (for fat oxidation rate (a)). *Significantly different between trials at the same time point, *P* ≤ 0⋅03 (for carbohydrate oxidation rate (b)).

The fat and carbohydrate oxidation rates throughout the trial are shown in Fig. 4. At baseline (09.00), there were no differences in fat or carbohydrate oxidation rates between the trials. A significant trial-by-time interaction was observed for fat oxidation rate (*P* = 0⋅005; Fig. 4(a)). *Post-hoc* tests revealed that the fat oxidation was higher in the low GI trial than in the high GI trial at 11.15 (95 % CI 0⋅006 to 0⋅1 g/min, *P* = 0⋅03, ES = 0⋅53). A significant trial-by-time interaction was observed for the carbohydrate oxidation rate (*P* < 0⋅001; Fig. 4(b)). *Post-hoc* tests revealed that the carbohydrate oxidation rates were lower in the low GI trial than in the high GI trial at 11.15, 11.30, 11.45, and 12.00 (95 % CI −0⋅3 to −0⋅04 g/min, *P* = 0⋅009, ES = 0⋅65; 95 % CI −0⋅2 to −0⋅02 g/min, *P* = 0⋅02, ES = 0⋅56; 95 % CI −0⋅1 to −0⋅02 g/min, *P* = 0⋅024, ES = 0⋅56; and 95 % CI −0⋅3 to −0⋅2 g/min, *P* = 0⋅03, ES = 0⋅55, respectively).

### Plasma glucose and insulin

**Fig. 5.**
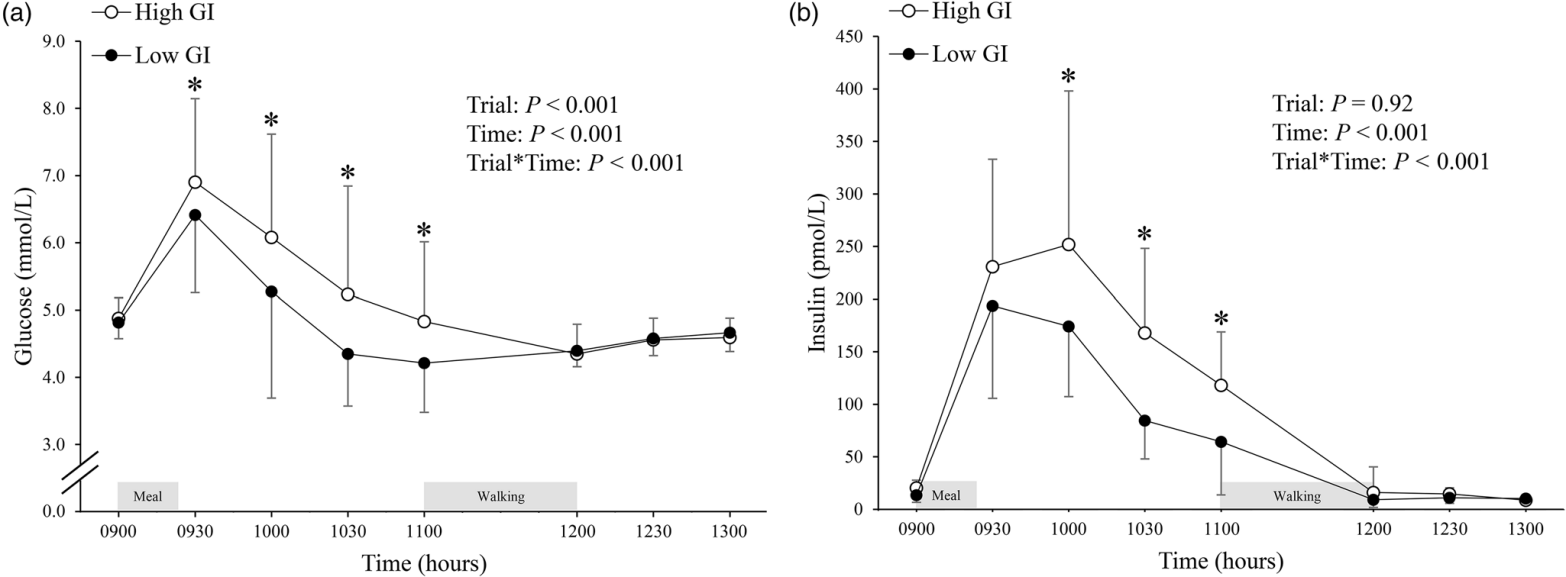
Glucose (a) and insulin (b) concentrations in the high and low glycaemic index (GI) trials. Values are means ± sd represented by unidirectional bars. Values were compared using generalised estimating equations. *Post-hoc* analysis was adjusted for multiple comparisons using the Bonferroni method. *Significantly different between trials at the same time point, *P* ≤ 0⋅04 (for glucose concentrations (a)). *Significantly different between trials at the same time point, *P* ≤ 0⋅02 (for insulin concentrations (b)).

The circulating concentrations of plasma glucose and insulin in each trial are shown in Fig. 5. There was no difference in the fasting (09.00) plasma glucose concentrations between the trials. There was a difference in fasting (09.00) plasma insulin concentrations between trials (95 % CI 2⋅7 to 11⋅3 pmol/l, *P* = 0⋅001, ES = 0⋅80). The main effect of trial and trial-by-time interactions was found for plasma glucose concentrations (both *P* < 0⋅001; Fig. 5(a)). *Post-hoc* tests of an interaction effect revealed that plasma glucose concentrations were higher in the high GI trial than in the low GI trial at 09.30, 10.00, 10.30, and 11.00 (95 % CI 0⋅3 to 17⋅3 mmol/l, *P* = 0⋅04, ES = 0⋅50; 95 % CI 8⋅6 to 20⋅4 mmol/l, *P* < 0⋅001, ES = 1⋅18; 95 % CI 5⋅0 to 26⋅9 mmol/l, *P* = 0⋅004, ES = 0⋅73; and 95 % CI 2⋅8 to 19⋅5 mmol/l, *P* = 0⋅009, ES = 0⋅67, respectively). A significant trial-by-time interaction was found for plasma insulin concentrations (*P* < 0⋅001; Fig. 5(b)). *Post-hoc* tests revealed that plasma insulin concentrations were higher in the high GI trial than in the low GI trial at 10.00, 10.30, and 11.00 (95 % CI 17⋅2 to 115⋅8 pmol/l, *P* = 0⋅008, ES = 0⋅62; 95 % CI 32⋅4 to 102⋅5 pmol/l, *P* < 0⋅001, ES = 1⋅15; and 95 % CI 5⋅2 to 65⋅6 pmol/l, *P* = 0⋅02, ES = 1⋅22, respectively). The total AUC over the postprandial period (09.00–11.00) for glucose (high GI: 11⋅5 ± 2⋅3 *v.* low GI: 10⋅3 ± 1⋅6 mmol/l⋅2 h, 95 % CI 0⋅8 to 1⋅8, *P* < 0⋅001, ES = 0⋅53) and insulin (high GI: 359⋅9 ± 153⋅4 *v.* low GI: 245⋅7 ± 77⋅1 pmol/l⋅2 h, 95 % CI 64⋅3 to 164⋅2 pmol/l⋅2 h, *P* < 0⋅001, ES = 1⋅12) concentrations were higher in the high GI trial than in the low GI trial.

### Serum TG, NEFA, glycerol, and 3-hydroxybutyrate

**Fig. 6.**
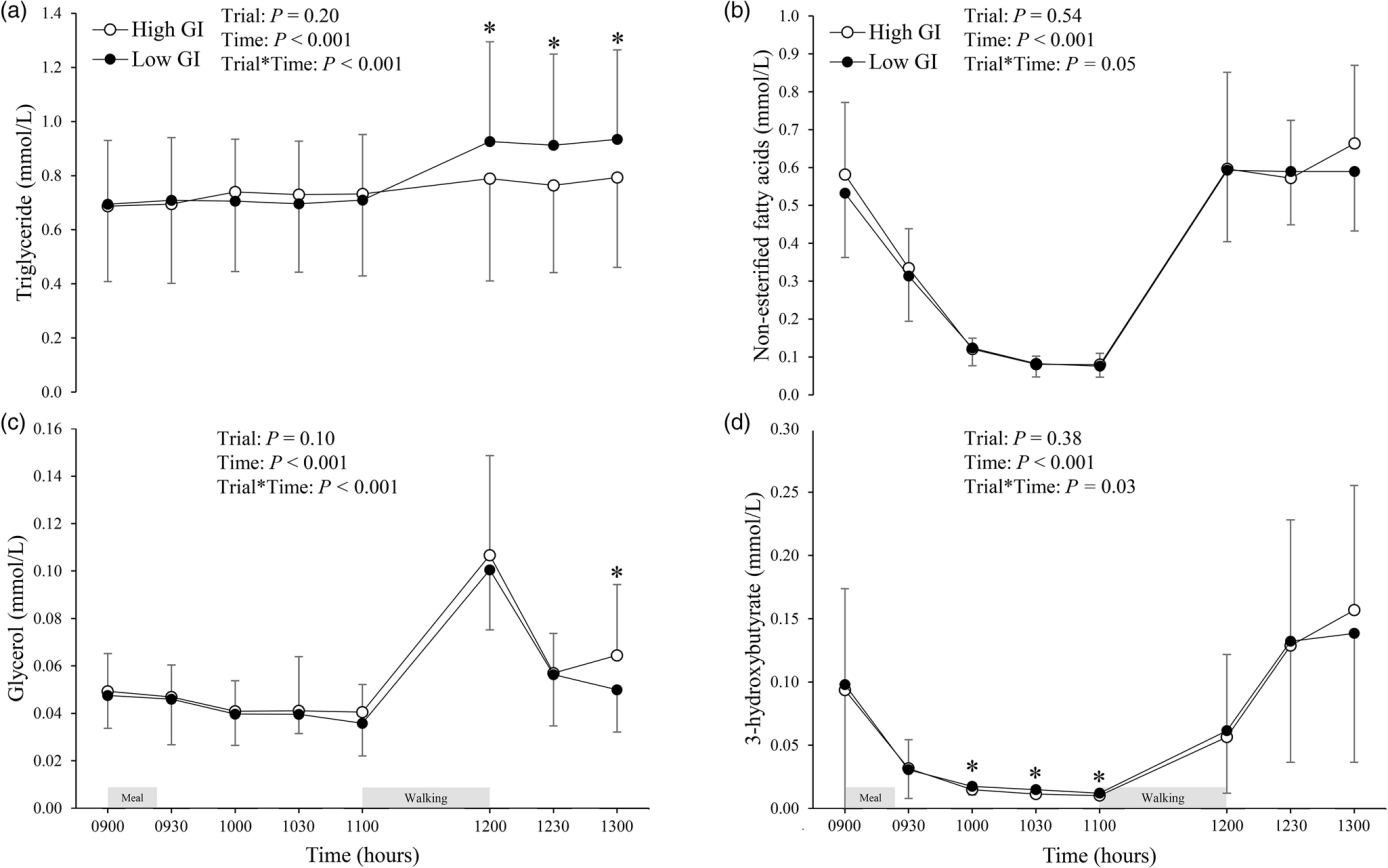
Triglyceride (a), non-esterified fatty acid (b), glycerol (c), and 3-hydroxybutyrate (d) concentrations in the high and low glycaemic index (GI) trials. Values are means ± sd represented by unidirectional bars. Values were compared using generalised estimating equations. *Post-hoc* analysis was adjusted for multiple comparisons using the Bonferroni method. *Significantly different between trials at the same time point, *P* < 0⋅001 (for triglyceride concentrations (a)). *Significantly different between trials at the same time point, *P* = 0⋅001 (for glycerol concentrations (c)). *Significantly different between trials at the same time point, *P* ≤ 0⋅02 (for 3-hydroxybutyrate concentrations (d)).

The circulating concentrations of serum TG, NEFA, glycerol, and 3-hydroxybutyrate in each trial are shown in Fig. 6. There were no differences in the fasting concentrations (09.00) of TG, NEFA, glycerol, or 3-hydroxybutyrate between the trials. A significant trial-by-time interaction was found for serum triglyceride concentration (*P* < 0⋅001; Fig. 6(a)). *Post-hoc* tests revealed that serum TG concentrations were higher in the low GI trial than in the high GI trial at 12.00, 12.30, and 13.00 (95 % CI 0⋅07 to 0⋅2 mmol/l, *P* < 0⋅001, ES = 1⋅05; 95 % CI 0⋅09 to 0⋅2 mmol/l, *P* < 0⋅001, ES = 1⋅16; and 95 % CI 0⋅08 to 0⋅2 mmol/l, *P* < 0⋅001, ES = 1⋅07, respectively). No trial-by-time interactions were found in serum NEFA concentrations (Fig. 6(b)). A significant trial-by-time interaction was observed for serum glycerol concentrations (*P* < 0⋅001; Fig. 6(c)). *Post-hoc* tests revealed that serum glycerol concentration was higher in the high GI trial than in the low GI trial at 13.00 (95 % CI 0⋅006 to 0⋅02 mmol/l, *P* = 0⋅001, ES = 0⋅79). A significant trial-by-time interaction was noted for serum 3-hydroxybutyrate concentrations (*P* = 0⋅03; Fig. 6(d)). *Post-hoc* tests revealed that serum 3-hydroxybutyrate concentrations were higher in the low GI trial than in the high GI trial at 10.00, 10.30, and 11.00 (95 % CI 0⋅0005 to 0⋅005 mmol/l, *P* = 0⋅02, ES = 0⋅33; 95 % CI 0⋅001 to 0⋅006 mmol/l, *P* = 0⋅001, ES = 0⋅63; and 95 % CI 0⋅0003 to 0⋅004 mmol/l, *P* = 0⋅02, ES = 0⋅23, respectively).

### Plasma total PYY and acylated ghrelin

The circulating concentrations of total plasma PYY and acylated ghrelin in each trial are shown in Supplementary Fig. S1. There were no differences in the fasting concentrations (09.00) of total PYY or acylated ghrelin between the trials. A main effect of trial and trial-by-time interaction was found for the total plasma PYY concentration (*P* = 0⋅04 and *P* < 0⋅001, respectively; Supplementary Fig. S1(a)). *Post-hoc* tests of an interaction effect revealed that plasma total PYY concentrations were higher in the low GI trial than in the high GI trial at 09.30 and 11.00 (95 % CI 0⋅01 to 0⋅08 ng/ml, *P* = 0⋅005, ES = 0⋅73 and 95 % CI 0⋅01 to 0⋅08 ng/ml, *P* = 0⋅009, ES = 0⋅43, respectively). A significant trial-by-time interaction was observed for the plasma-acylated ghrelin concentration (*P* = 0⋅004; Supplementary Fig. S1(b)). Subsequent *post-hoc* tests did not reveal between-trial differences in plasma acylated ghrelin concentrations.

### Food reward

Explicit liking, explicit wanting, implicit wanting, and relative preferences for fat appeal and taste appeal biases in each trial are shown in Table 2. There were no differences in fasting values (09.00) for all parameters except for implicit wanting and relative preference for taste appeal bias (95 % CI 0⋅06 to 14⋅2, *P* = 0⋅05, ES = 0⋅75 and 95 % CI 1⋅6 to 6⋅3, *P* < 0⋅001, ES = 0⋅81, respectively) between trials. No main effects of the trial were found in explicit liking, explicit wanting, implicit wanting, and relative preference for fat appeal bias, or explicit liking and explicit wanting for taste appeal bias. Implicit wanting and relative preference for taste appeal bias were higher in the high GI trial than in the low GI trial (95 % CI 1⋅6 to 12⋅6, *P* = 0⋅03, ES = 0⋅40 and 95 % CI 1⋅0 to 5⋅2, *P* = 0⋅03, ES = 0⋅44; a main effect of the trial). Trial-by-time interactions were not observed for any parameters.

**Table 2.** Explicit liking, explicit wanting, implicit wanting, and relative preference of fat appeal bias and taste appeal bias in the high and low glycaemic index (GI) trials

Time (hours)	09.00		10.00		13.00	
	High GI	Low GI	High GI	Low GI	High GI	Low GI
Fat appeal bias						
Explicit liking	−12⋅6 ± 12⋅4	−12⋅4 ± 12⋅4	−4⋅7 ± 11⋅6	−2⋅2 ± 8⋅6	−3⋅9 ± 12⋅8	−1⋅6 ± 7⋅7
Explicit wanting	−16⋅0 ± 14⋅1	−16⋅7 ± 16⋅9	−6⋅3 ± 8⋅8	−2⋅2 ± 11⋅1	−6⋅0 ± 13⋅8	−4⋅0 ± 10⋅0
Implicit wanting	−27⋅6 ± 29⋅9	−28⋅7 ± 28⋅0	−28⋅0 ± 28⋅1	−17⋅9 ± 23⋅9	−12⋅1 ± 29⋅0	−5⋅2 ± 27⋅6
Relative preference	−12⋅5 ± 13⋅6	−11⋅5 ± 11⋅6	−11⋅5 ± 12⋅1	−6⋅7 ± 8⋅9	−5⋅1 ± 12⋅2	−2⋅1 ± 11⋅7
Taste appeal bias						
Explicit liking	−2⋅8 ± 16⋅1	2⋅1 ± 14⋅4	6⋅3 ± 12⋅4	10⋅1 ± 16⋅1	−5⋅3 ± 17⋅0	−5⋅5 ± 16⋅2
Explicit wanting	−1⋅2 ± 17⋅4	0⋅1 ± 13⋅8	8⋅6 ± 12⋅3	9⋅9 ± 15⋅8	−6⋅7 ± 19⋅3	−5⋅8 ± 15⋅9
Implicit wanting*	−4⋅5 ± 44⋅0	2⋅6 ± 43⋅7	7⋅5 ± 42⋅7	23⋅3 ± 40⋅9	−18⋅3 ± 43⋅2	−17⋅0 ± 44⋅2
Relative preference*	−2⋅1 ± 16⋅8	1⋅9 ± 16⋅6	3⋅9 ± 16⋅9	8⋅9 ± 16⋅7	−7⋅0 ± 17⋅3	−6⋅5 ± 17⋅6

Values are means ± sd. Values were compared using generalised estimating equations.

* Significantly different between trials, a main effect of trial, *P* = 0⋅03.

### Subjective appetite

The subjective appetites for each trial are listed in Table 3. There were no differences in fasting values (09.00) for all parameters except satiety (*P* = 0⋅05). No significant effects of the trial were observed for any of the parameters. Trial-by-time interactions were not observed for satiety. A significant trial-by-time interaction was observed for fullness (*P* = 0⋅002). *Post-hoc* tests revealed that fullness was higher in the low GI trial than in the high GI trial at 13.00 (95 % CI 0⋅02 to 18⋅8, *P* = 0⋅049, ES = 0⋅49). Significant trial-by-time interactions were found between prospective food intake and subjective appetite scores (both *P* < 0⋅001). Subsequent *post-hoc* tests did not reveal where the between-trial differences were for prospective food intake or subjective appetite scores.

**Table 3.** Subjective appetite in the high and low glycaemic index (GI) trials

Time (hours)		09.00	09.30	10.00	10.30	11.00	12.00	12.30	13.00
Satiety (mm)	High GI	15⋅2 ± 17⋅3	75⋅3 ± 20⋅4	70⋅6 ± 23⋅2	68⋅7 ± 22⋅1	55⋅6 ± 26⋅4	54⋅7 ± 29⋅2	36⋅5 ± 27⋅4	32⋅4 ± 28⋅0
	Low GI	23⋅6 ± 20⋅6	75⋅3 ± 25⋅8	75⋅7 ± 19⋅3	71⋅0 ± 19⋅6	62⋅2 ± 26⋅5	45⋅8 ± 29⋅3	38⋅1 ± 25⋅5	31⋅7 ± 23⋅4
Fullness (mm)	High GI	13⋅3 ± 17⋅5	78⋅8 ± 20⋅9	76⋅6 ± 22⋅6	69⋅9 ± 24⋅0	64⋅8 ± 24⋅3	51⋅8 ± 29⋅1	39⋅2 ± 26⋅4	24⋅1 ± 18⋅0
	Low GI	14⋅5 ± 18⋅7	84⋅7 ± 12⋅3	76⋅4 ± 16⋅6	73⋅5 ± 19⋅1	59⋅1 ± 25⋅2	50⋅3 ± 29⋅5	38⋅6 ± 30⋅6	33⋅6 ± 23⋅6*
Hunger (mm)	High GI	82⋅1 ± 14⋅5	18⋅7 ± 24⋅6	21⋅5 ± 21⋅6	27⋅8 ± 25⋅6	31⋅6 ± 24⋅1	40⋅8 ± 30⋅0	59⋅3 ± 28⋅9	71⋅0 ± 24⋅0
	Low GI	76⋅0 ± 15⋅1	14⋅0 ± 16⋅5	17⋅8 ± 19⋅6	28⋅7 ± 25⋅5	42⋅8 ± 27⋅0	54⋅4 ± 30⋅6	60⋅7 ± 27⋅9	71⋅1 ± 20⋅7
Prospective food intake (mm)	High GI	70⋅5 ± 20⋅4	35⋅8 ± 29⋅7	32⋅5 ± 26⋅7	36⋅4 ± 29⋅1	39⋅8 ± 28⋅1	44⋅6 ± 29⋅7	59⋅6 ± 27⋅2	73⋅9 ± 21⋅4
	Low GI	68⋅9 ± 22⋅6	30⋅5 ± 23⋅6	39⋅5 ± 25⋅7	43⋅9 ± 25⋅6	48⋅8 ± 26⋅8	50⋅8 ± 32⋅4	67⋅5 ± 25⋅5	73⋅0 ± 22⋅2
Subjective appetite score	High GI	19⋅0 ± 14⋅6	74⋅9 ± 21⋅4	73⋅3 ± 22⋅5	68⋅6 ± 23⋅1	62⋅3 ± 22⋅3	55⋅3 ± 25⋅8	39⋅2 ± 26⋅0	27⋅9 ± 19⋅3
	Low GI	29⋅6 ± 16⋅0	81⋅7 ± 15⋅7	76⋅4 ± 18⋅9	70⋅4 ± 19⋅6	59⋅9 ± 25⋅7	51⋅2 ± 28⋅2	40⋅5 ± 24⋅9	33⋅6 ± 22⋅2

Values are means ± sd. Values were compared using generalised estimating equations and *post-hoc* analysis was adjusted for multiple comparisons using the Bonferroni method. Analysis revealed an interaction effect for fullness (*P* = 0⋅002).

* Significantly different from the high GI trial at the same time point, *P* = 0⋅049 (i.e. *post-hoc* analysis of an interaction effect).

## Discussion

The present study demonstrated that consuming a low GI meal increased fat oxidation and decreased carbohydrate oxidation compared to a prior high GI meal during a 60-min walk in middle-aged women. Despite these changes in substrate oxidation during walking and postprandial alterations in glucose, insulin, and total PYY concentrations, consuming low GI meals did not appear to have a significant overall effect on homeostatic or non-homeostatic appetite assessed over time.

In the present study, fat oxidation was enhanced and carbohydrate oxidation was suppressed during exercise when a low GI meal was consumed 100 min before performing low intensity exercise. These findings observed in ‘middle-aged’ women coincide with most of the previous acute studies conducted in young men^(13–17,19,20,22–24)^ and women.^(18,21)^ The increase in fat oxidation during exercise observed in the low GI trial may have been mediated by several mechanisms. First, lower elevations in plasma glucose and insulin concentrations in the postprandial period may have influenced fat utilisation during subsequent exercise. A previous study reported that an increase in plasma insulin concentration before exercise reduces lipolysis and limits fat oxidation during exercise.^(51)^ This was confirmed by the elevated insulin concentrations observed during the postprandial period in a high GI trial. Another potential mechanism is that differences in GI may affect muscle glycogen synthesis and utilisation.^(44)^ It has been reported that muscle glycogen concentrations increase after a high GI meal but remain unchanged after a low GI meal, and muscle glycogen utilisation during exercise is greater in high GI trials than in low GI trials.^(52)^ Although this previous study^(52)^ employed trained men and participants consumed a larger amount of carbohydrates (i.e. 2⋅5 g/kg body mass *v.* 1⋅5 g/kg body mass of the present study) and performed higher-intensity exercise (i.e. running at 71 % of maximum oxygen uptake for 30 min *v.* walking at 50 % of estimated maximum oxygen uptake for 60 min of the present study), postprandial muscle glycogen content and its utilisation during exercise may have been lowered after consuming the low GI meal in the present study, which may have resulted in lower carbohydrate oxidation during exercise in the low GI trial than in the high GI trial. It should be emphasised that these findings are more important for elderly women who exercise for health because the decline in fat-free mass with age is related to a decrease in basal fat oxidation,^(28,29)^ and these alterations may contribute to the development of risk factors for cardiovascular disease. Further studies should explore whether the findings of this acute study can be applied to older women over an extended period.

To the best of our knowledge, the present study is the first to examine the acute effects of prior GI meals on substrate oxidation during exercise in Asian (Japanese) women, as most studies examining this area have employed participants from Western countries (for a review, see^(5)^), with only limited studies in Asia.^(24)^ It is important to highlight that the cumulative (total) amount of carbohydrate oxidised during a 60-min walk in both trials was smaller in the present study than in the previous study conducted in the UK with a similar study design,^(21)^ despite the fact that a larger amount of carbohydrate was provided in this study (i.e. 1⋅5 g/kg body mass) compared with the previous study (1⋅0 g/kg body mass). Although it is difficult to make a direct comparison between studies because of the different intensities of exercise, test meal content and postprandial periods prior to exercise, these differences may be explained by the magnitude and/or patterns of postprandial glucose and insulin responses. The duration of postprandial glucose elevation in both trials was shorter than that in a previous study.^(21)^ Furthermore, the magnitude and duration of the postprandial insulin elevation in both trials were smaller than those reported in a previous study.^(21)^ Indeed, some evidence suggests that East Asians have better insulin sensitivity and lower insulin secretory capacity than Caucasians and Africans.^(53)^ Collectively, these ethnic differences may affect substrate oxidation during exercise, partly due to different postprandial metabolic responses. Therefore, the contents of the test meal and the timing of pre-exercise GI meals, in particular, may need to be considered according to ethnicity.

In the present study, serum 3-hydroxybutyrate concentrations during the last hour of the postprandial period were higher in the low GI trial than in the high GI trial. This finding suggests that consuming a low GI meal could have promoted the catabolism of fatty acids because 3-hydroxybutyrate is synthesised from acetyl-coenzyme A, which is produced by the oxidation of fatty acids in the liver.^(54)^ The reduced postprandial insulin observed in the low GI trial, as high insulin inhibits fatty acid mobilisation,^(55)^ may be a possible reason for this finding, although this is difficult to ascertain because differences were not observed in the concentrations of serum TG, NEFA, and glycerol during the postprandial period between trials. It is worth noting that elevated serum TG concentrations, which have not been examined in previous studies on pre-exercise GI meals and substrate oxidation,^(5)^ were observed in the low GI trial compared to the high GI trial during the post-exercise period in the present study. This may be due to the enhanced removal of TG from the adipose tissue through elevated fat oxidation during walking in the low GI trial rather than exogenous TG from the meal, as a similar elevation of postprandial TG responses was observed prior to isoenergetic walking in both trials. However, these potential explanations should be interpreted with caution because we were unable to assess TG kinetics (i.e. rates of appearance and disappearance) in the present study, which makes it difficult to interpret the findings of elevated glycerol concentrations observed at the end of the low GI trial.

Several studies have reported that the hedonic system of non-homeostatic appetite control is affected by meal-induced changes in hormone concentrations, habitual physical activity, and structured exercise.^(34,56–59)^ A previous study suggested that insulin secretion decreases dopamine signalling, which could cause a decrease in the rewarding aspects of food.^(34)^ Additionally, the stimulation of ghrelin has been reported to activate reward-related areas in the brain, such as the amygdala, ventral striatum, anterior insula, orbitofrontal cortex, and hippocampus.^(58)^ Furthermore, a previous study conducted in young men showed lower reactivity to high-density energy foods than to low-density energy foods using blood oxygen level-dependent functional magnetic resonance imaging after acute high intensity exercise.^(59)^ In the present study, there were significant differences in implicit wanting and the relative preference for taste appeal bias between the high and low GI trials. This result indicates that consuming a low GI meal may suppress the preference for sweet foods. However, given the lack of trial-by-time interaction observed in the present study, this finding should be interpreted cautiously. Although the trial effect was observed even after adjusting for baseline values as a covariate, it is unknown whether the elevated implicit wanting and relative preference for taste appeal bias observed in the low GI trial were influenced by different GI meals, exercise, or a combination of both. Thus, further studies are required to clarify the effects of meals with different GIs and exercises on food reward.

Regarding homeostatic appetite, the findings of enhanced total PYY concentrations during the postprandial period in the low GI trial compared to the high GI trial in the present study are not consistent with those of a previous study that reported no difference in total PYY concentrations between low and high GI trials in young, sedentary women.^(21)^ The elevated total PYY concentrations observed in the low GI trial in the present study may be explained by the fact that carbohydrates in the low GI meal reached the distal small intestine and stimulated intestinal L cells (i.e. both PYY and glucagon-like peptide-1 were co-localised and co-secreted from L cells) compared with the high GI meal.^(60)^ However, this speculation highlights the need for caution as the present study did not evaluate directly the mechanistic roles of the meal-induced satiety hormone response. Regarding subjective appetite, while some previous studies have shown that fullness was higher in the low GI trial than in the high GI trial during the postprandial period,^(17,18)^ there were no differences between trials in the present study. A possible reason for this is that the difference in the amounts of fibre between the high and low GI meals was smaller than that in previous studies,^(17,18)^ which would have affected the difference in fullness. Although fullness was higher in the low GI trial than in the high GI trial only at the end of the experiment (i.e. 13.00) in the present study, the significance of this finding is uncertain, as there were no differences in other subjective appetite parameters (satiety, hunger, prospective food intake, and subjective appetite score) or appetite-related hormones (i.e. acylated ghrelin and total PYY) between trials in the present study. Therefore, the effects of a low GI diet on these parameters, along with the actual food intake after exercise, need to be carefully examined in future longitudinal studies.

The present study had several strengths. Middle-aged women were employed, whereas most previous studies examining the effects of the different GI meals on substrate oxidation during subsequent exercise have employed young men, with few studies in middle-aged or older women (for a review of these, see^(5)^). Additionally, our study was conducted on Asian (Japanese) women. The population and ethnicity recruited in our study are important because an exaggerated postprandial glycaemic excursion has been observed with increasing age,^(61)^ and Asians have impaired insulin sensitivity and β-cell function compared to white Europeans (for a review of these, see^(25)^). Moreover, the present study is the first to evaluate non-homeostatic appetite (i.e. food reward) after consuming high and low GI meals. The reward system for food affects chronic eating behaviour and thus has the potential to support long-term self-management of health. A notable limitation is the inability to predict glycaemic responses to mixed meals, in which the GI of the total food item was calculated from the constituent foods.^(44)^ The estimated GI ratio of the two test meals was 1⋅78:1⋅00 (i.e. the GI values were 73 and 41 for the high and low GI meals, respectively), and the actual measured ratio of the incremental AUC for glucose curve was 1⋅31:1⋅00. Additionally, female sex hormones, including estrogen and progesterone, were not assessed in the present study, which may have inaccurately determined the phase of the menstrual cycle. Moreover, the present study did not include a non-exercise control condition and this makes us to interpret the findings difficult for distinguishing between the meal and exercise effects. Finally, we only recruited a single ethnic group. The direct ethnic comparisons may help to propose the ideal timing of post-meal exercise, assuming different postprandial metabolic responses among ethnicities.^(62)^

In conclusion, the present study shows that consuming a low GI meal elicits enhanced fat oxidation during a subsequent 60-min walk in middle-aged women. However, differences in postprandial glucose and insulin concentrations induced by different GI meals did not affect subjective appetite or food reward evaluated over time in the present study.

## Supporting information

Sakazaki et al. supplementary material 1 Sakazaki et al. supplementary material

Sakazaki et al. supplementary material 2 Sakazaki et al. supplementary material

## Supplementary material

The supplementary material for this article can be found at https://doi.org/10.1017/jns.2023.96.
