# Supplementary material for: Effects of pre-exercise high and low glycaemic index meals on substrate metabolism and appetite in middle-aged women
Source: J Nutr Sci. 2023 Nov 20;12:e114. doi: 10.1017/jns.2023.96 (PMC10660074; doi:10.1017/jns.2023.96)
Supplement: Sakazaki et al. supplementary material 1 — Sakazaki et al. supplementary material [file S2048679023000964sup001.pdf]

Title: Effects of pre-exercise high and low glycaemic index meals on substrate metabolism and appetite in middle-aged women

Authors: Miki Sakazaki, et al.

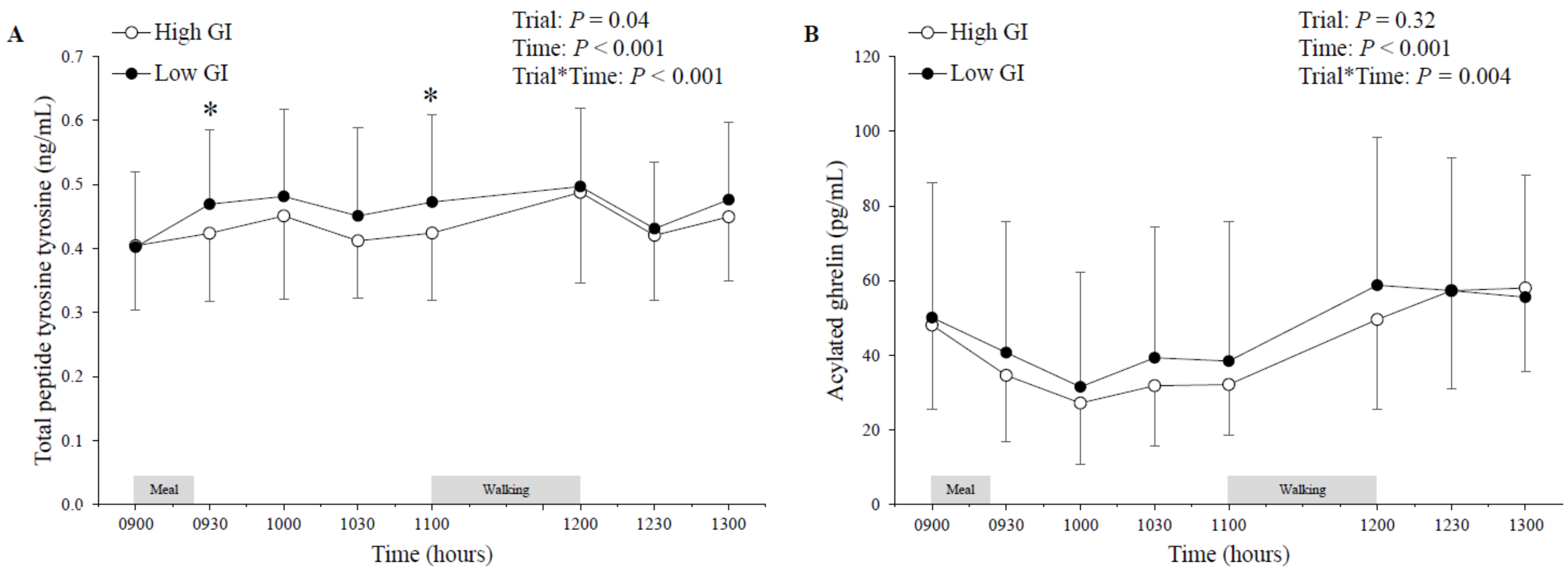

SUPPLEMENTAL FIGURE 1 Total peptide tyrosine tyrosine (A) and acylated ghrelin (B) concentrations in the high and low glycaemic index (GI) trials

GI, glycaemic index. Values are means ± standard deviation represented by unidirectional bars. Values were compared using generalised estimating equations. Post-hoc analysis was adjusted for multiple comparisons using the Bonferroni method. *Significantly different between trials, $P \leq 0.009$ (for total peptide tyrosine tyrosine concentrations (A)).
