# Supplementary material for: Effects of pre-exercise high and low glycaemic index meals on substrate metabolism and appetite in middle-aged women
Source: J Nutr Sci. 2023 Nov 20;12:e114. doi: 10.1017/jns.2023.96 (PMC10660074; doi:10.1017/jns.2023.96)
Supplement: Sakazaki et al. supplementary material 2 — Sakazaki et al. supplementary material [file S2048679023000964sup002.pdf]

Title: effects of pre-exercise high and low glycaemic index meals on substrate metabolism and appetite in middle-aged women

Authors: Miki Sakazaki, et al.

SUPPLEMENTAL TABLE 1 Sixteen food photographs, four food images for each of the four food categories

**Category**	**High-fat savoury**	**Low-fat savoury**	**High-fat sweet**	**Low-fat sweet**
**Food image**	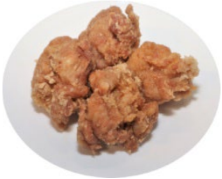<br>Fried chicken	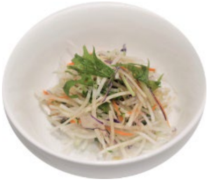<br>Radish salad	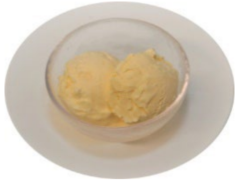<br>Vanilla ice cream	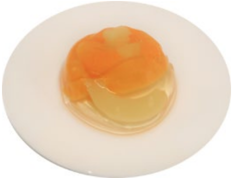<br>Fruit jelly
	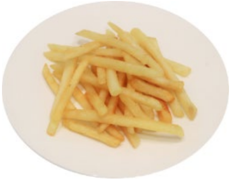<br>French fries	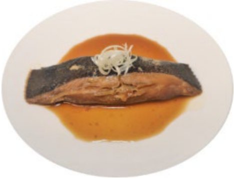<br>Boiled flounder	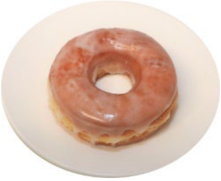<br>Doughnuts	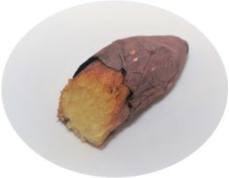<br>Baked potato
	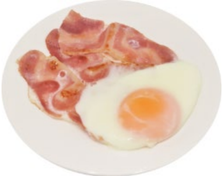<br>Bacon and eggs	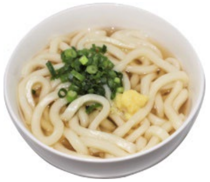<br>Udon noodles	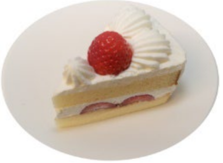<br>Strawberry cake	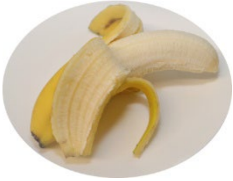<br>Banana
	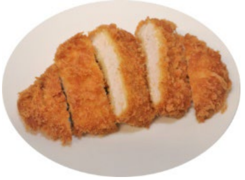<br>Pork cutlet	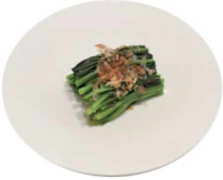<br>Boiled spinach	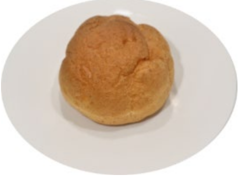<br>Cream puffs	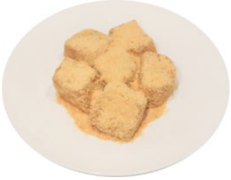<br>Bracken rice cake
